# miRNAs As Emerging Regulators of Oligodendrocyte Development and Differentiation

**DOI:** 10.3389/fcell.2016.00059

**Published:** 2016-06-17

**Authors:** Dylan A. Galloway, Craig S. Moore

**Affiliations:** Division of BioMedical Sciences, Faculty of Medicine, Memorial University of NewfoundlandSt. John's, NL, Canada

**Keywords:** oligodendrocyte, miRNA, myelination, multiple sclerosis, leukodystrophy

## Abstract

Chronic demyelination is a hallmark of neurological disorders such as multiple sclerosis (MS) and several leukodystrophies. In the central nervous system (CNS), remyelination is a regenerative process that is often inadequate during these pathological states. In the MS context, *in situ* evidence suggests that remyelination is mediated by populations of oligodendrocyte progenitor cells (OPCs) that proliferate, migrate, and differentiate into mature, myelin-producing oligodendrocytes at sites of demyelinated lesions. The molecular programming of OPCs into mature oligodendrocytes is governed by a myriad of complex intracellular signaling pathways that modulate this process. Recent research has demonstrated the importance of specific and short non-coding RNAs, known as microRNAs (miRNAs), in regulating OPC differentiation and remyelination. Fortunately, it may be possible to take advantage of numerous developmental studies (both human and rodent) that have previously characterized miRNA expression profiles from the early neural progenitor cell to the late myelin-producing oligodendrocyte. Here we review much of the work to date and discuss the impact of miRNAs on OPC and oligodendrocyte biology. Additionally, we consider the potential for miRNA-mediated therapy in the context of remyelination and brain repair.

## Introduction

Myelination of neurons is a critical process required for normal functioning of the adult mammalian central nervous system (CNS). A loss of myelin (i.e., demyelination) is a pathological hallmark in multiple sclerosis (MS) and several leukodystrophies, and significantly contributes to the clinical manifestations of such diseases. Further understanding the cellular and molecular mechanisms responsible for promoting and/or enhancing myelination within the CNS may provide novel therapeutic avenues for those suffering from demyelinating disorders. While several independent factors contribute to both the processes of developmental myelination and remyelination in the CNS, many of these factors affect the highly regulated transition from an immature proliferating oligodendrocyte progenitor cell (OPC) into a mature and functional myelinating oligodendrocyte (OL). Over the past decade, one of the molecular mechanisms that has received growing interest is the role of microRNAs (miRNAs), short non-coding RNA molecules, during OL development and myelination (Barca-Mayo and Lu, 2012). Herein, we will review the emerging function of miRNAs and their influences on CNS myelin development and remyelination during brain repair.

### Oligodendrocyte development in the CNS

The ontogeny of OL development is complex and controlled by multiple extrinsic and intrinsic mechanisms that promote the progression from a pluripotent progenitor cell to an OL, many of which are reviewed elsewhere (Miller, 2002; Emery, 2010; Gallo and Deneen, 2014). Throughout this process, OPC proliferation and differentiation are differentially regulated, with the cessation in OPC proliferation being linked to OL differentiation and maturation. This phenomenon is similar to those observed in a variety of different post-mitotic cell types, however inhibiting cell cycle progression is not sufficient to induce OL differentiation (Casaccia-Bonnefil and Liu, 2003). Thus, the coordination of multiple signaling pathways is needed for progenitor cells to move from an immature state to a functional OL, a process that is achieved in part by miRNAs.

### miRNAs: biogenesis and function

miRNAs are endogenous, small non-coding RNA molecules, typically 20–22 base pairs in length, and regulate gene expression by binding to the 3′ untranslated region (UTR) of targeted mRNA molecules. Through this process, miRNAs can regulate hundreds of target genes and influence multiple pathways involved in cellular development and homeostasis (Bartel, 2004; Winter et al., 2009). Due to the critical roles of miRNAs, their production and function is highly regulated within cells. The canonical biogenesis pathway is initiated by the transcription of the primary miRNA transcript, the pri-miRNA. This transcript is cleaved within the nucleus by the Drosha-DGCR8 complex, thus forming the precursor hairpin miRNA or pre-miRNA. The pre-miRNA is transported into the cytoplasm via Exportin-5 - Ran-GTP nuclear export. Within the cytoplasm the RNase enzyme Dicer1, along with the RNA binding protein TRBP, then cleaves the pre-miRNA to form the mature miRNA duplex consisting of both the -5p and -3p mature miRNAs. Mature miRNAs associate with Ago2 protein to form the RNA-induced silencing complex (RISC), which binds mRNA within the cytoplasm and ultimately leads to cleavage, repression or deadenylation of the target mRNA. Within the genome, many miRNAs aggregate within the same pri-miRNA transcript, resulting in a miRNA family or cluster. The clustering of miRNAs has functional significance and allows multiple miRNAs with similar gene targets or function to be transcribed simultaneously (Altuvia et al., 2005).

### miRNAs as emerging regulators of CNS demyelination

Due to the broad actions of miRNAs in controlling multiple systems (e.g., cell differentiation, apoptosis, inflammation, homeostasis), it is unsurprising that miRNAs have emerged as important molecules that regulate a variety of pathogenic mechanisms in demyelinating diseases, including MS, leukodystrophies, and perinatal hypoxia-ischemia. The most well-characterized human demyelinating disease is MS, a chronic inflammatory disease that affects the CNS. Although largely immune-mediated, there is growing evidence suggesting primary impairments in oligodendrocyte biology or an oligodendropathy (Kutzelnigg et al., 2005; Trapp and Nave, 2008). miRNAs are key regulators in MS pathology, as well as its animal model experimental autoimmune encephalomyelitis (EAE), although the primary focus has been on regulating the immune system. Beyond the immune system, studies highlighting miRNAs as key regulators of OPC differentiation and OL function may have significant clinical implications with respect to further understanding MS disease pathogenesis and identifying novel drug targets that can promote brain repair. Within this review, we will discuss several miRNAs that are specifically implicated in oligodendrocyte biology and myelination, many of which are altered in MS and other demyelinating diseases.

## miRNAs as key regulators of oligodendrocyte development

### Dicer1

Dicer is a critical miRNA-processing enzyme encoded by the *Dicer1* gene in mice and is required to generate functional mature miRNAs from miRNA precursors. Mice lacking a functional *Dicer1* gene are embryonically lethal (Bernstein et al., 2003), and as such, conditional knockouts are utilized to identify the requirement and/or roles of miRNAs in specific cell lineages and developmental studies. Approximately 70% of known miRNAs are expressed in the brain (Cao et al., 2006), suggesting the importance of miRNAs in the mammalian CNS. The role for miRNAs during OL development and myelination was first demonstrated in conditional knockouts lacking a functional Dicer enzyme in mature oligodendrocytes expressing proteolipid protein (PLP). This initial research led to the conclusion that these mice experienced a substantial CNS impairment (e.g., ataxia, paralysis) and did not survive beyond postnatal month 14 (Shin et al., 2009). Further analysis of these mice demonstrated reductions in several myelin proteins, including myelin associated glycoprotein (MAG), myelin oligodendrocyte protein (MOG), 2′,3′-Cyclic-nucleotide 3′-phosphodiesterase (CNPase) and PLP. The reduction in myelination was also accompanied by axonal amyloid precursor protein deposition, reduced axonal transport, increased oxidative stress, astrogliosis, and microglial activation. As this cre/loxP model led to reduced, albeit not abolished, *Dicer1* expression in OLs, a microarray analysis was performed using RNA isolated from whole brain. When compared to wildtype mice, transgenic mice had reduced expression of miR-32, -144, and -219, and upregulation of miR-7a, -7b, -181a-1, and -592. Following this initial finding, two concurrent reports demonstrated the necessity of miRNAs through conditional knockout of *Dicer1* in earlier OPC lineage cells (e.g., *Olig1, Olig2, CNP*) on OL development (Dugas et al., 2010; Zhao et al., 2010). Olig1 and Olig2 are highly expressed within OPCs, while CNP expression is highest during the differentiation of immature OLs (Zhou et al., 2000; Baumann and Pham-Dinh, 2001). All OL lineage conditional knockout mice presented a “shiverer” mouse-like (MBP KO) phenotype in early postnatal days, while *Olig1* and *CNP* targeted deletion led to mortality within 1 month. Interestingly, *Olig2* floxed mice survived, eventually generated normal appearing myelin, and were indistinguishable from wildtype littermate controls. Within the first postnatal month, all knockout mice showed reduced myelination, however, changes in axonal survival were not seen. Subsequent *in vivo* and *in vitro* studies using *CNP* and *Olig1* targeted *Dicer1* knockout mice demonstrated increased numbers of progenitor cells, suggesting a failure of OPCs to differentiate into mature OLs, and presents further evidence that miRNAs are indispensable for the transition from progenitor cell to myelinating OL.

### miR-219, -338, -138

**Figure 1:**
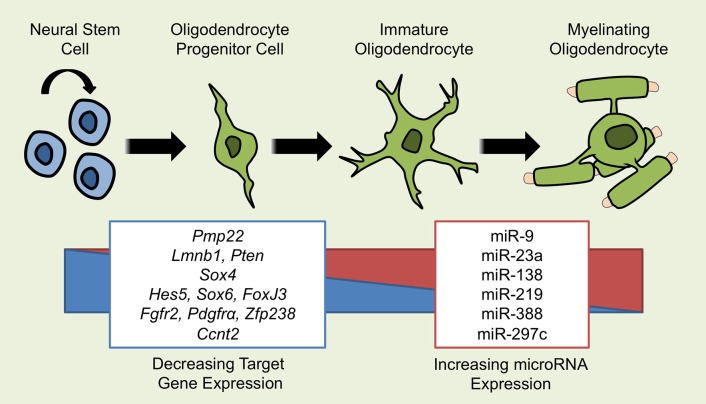
**miRNAs Implicated in OL Differentiation**. Several miRNAs have been demonstrated to influence the development from a self-renewing neural stem cell (NSC) to a mature, myelinating oligodendrocyte. As OLs differentiate, the expression of multiple miRNAs including miR-9, -23a, -138, -219, -338, and -297c increases and affects the process of differentiation through the repression of target gene mRNA. miRNAs that promote OL differentiation do so by inhibiting repressors of OL differentiation such as *Hes5, Sox6, Ccnt2*, and *Foxj3* or by inhibiting genes that promote the proliferation of NSCs and OPCs such as *Fgfr2* and *pdgfr*α. miRNAs may also exert effects through the suppression of inappropriate genes, as is the case for miR-9 suppressing *Pmp22*.

An analysis of miRNA profiles in normal CNS development and *Dicer1* knockout models has identified miR-219 as a critical miRNA involved in oligodendrocyte development (Shin et al., 2009; Dugas et al., 2010; Zhao et al., 2010) and has become a highly studied miRNA in the context of myelin biology. Microarray analysis of GalC^+^A2B5^−^ oligodendrocytes vs. A2B5^+^GalC^−^ neuronal progenitor cells identified elevated miR-219 expression in mature oligodendrocytes (Lau et al., 2008). Later reports show that miR-219 is a potent inducer of OL differentiation, while exogenous administration of miR-219 rescues deficits in myelination seen in *Dicer1* knockouts both *in vitro* and *in vivo* (Dugas et al., 2010; Zhao et al., 2010). The mechanism of action of miR-219 has been attributed to repressing factors that inhibit oligodendrocyte differentiation, including *Sox6, Hes5, Foxj3, Pdgfr-*α, and *Zfp238*. miR-219 expression remains elevated following differentiation to OL (Figure 1). The functional significance of elevated miR-219 expression has been attributed to the repression of *Elovl7*, a fatty acid elongation factor that can result in lipid accumulation and neurodegeneration when overexpressed (Shin et al., 2009). miR-219 expression is also upregulated during human OPC differentiation and correlates with the expression of PLP in human samples (de Faria et al., 2012). Within the adult human brain, miR-219 is highly expressed in OLs, with reduced but detectable expression in human A2B5^+^ and O4^+^ progenitors (Leong et al., 2014). Thus, miR-219 is not only important for the transition of OPC to OL, but may also be important in the formation and maintenance of myelin.

In addition to miR-219, miR-338 is also upregulated in mature vs. immature oligodendrocytes (Lau et al., 2008). miR-338 is highly expressed in the spinal cord and optic nerve, where it overlaps with oligodendrocyte markers CC1 and Olig2 (Zhao et al., 2010). Inhibition of miR-338 blocks oligodendrocyte maturation *in vitro*, while elevated miR-388 increases OL differentiation (Zhao et al., 2010). miR-338 also controls the differentiation of OPCs into mature oligodendrocytes through the inhibition of oligodendrocyte differentiation inhibitors, notably *Hes5* and *Sox6* (Zhao et al., 2010). Using *in silico* analysis, both miR-338 and miR-219 are predicted to target other genes including *NeuroD1, Isl1*, and *Otx2*, which are inhibitors of the transition from neural stem cell to OPC (Lee et al., 1995; Pfaff et al., 1996; Vernay et al., 2005; Zhao et al., 2010). In humans, miR-338 is upregulated during differentiation of human OPCs (de Faria et al., 2012). Furthermore, miR-338 is also present in neurons and targets enzymes involved in neurosteroid production (ARK1C1 and ARK1C2; Noorbakhsh et al., 2011), a finding that suggests additional roles for miR-338 in neural cell development beyond the OL lineage.

miR-138 is an additional miRNA with higher expression in mature oligodendrocytes compared with OPCs (Dugas et al., 2010). miR-138 expression is increased in both CNS white matter and oligodendrocytes, and has low expression in neurons and astrocytes. Notably, miR-138 is not as potent as other OL-promoting miRNAs such as miR-219 and miR-338 (Dugas et al., 2010). *In vitro*, miR-138 induced oligodendrocyte differentiation and expression of CNP and MBP. Additionally, miR-138 targets the *Sox4* transcription factor, a repressor of OL maturation (Potzner et al., 2007; Yeh et al., 2013). In contrast, sustained expression of miR-138 eventually leads to reductions in MOG protein, suggesting miR-138 may have a transient role in OPC differentiation and a Janus role in the transition to a mature OL.

Strikingly, miR-219 and -338 are two of the most highly downregulated miRNAs in chronic MS lesions (Junker et al., 2009). Due to the critical role of miR-219 and -338 in oligodendrocyte differentiation, this finding suggests a failure of OPCs to differentiate into mature OLs in chronic MS lesions. It is also possible that reductions in levels of these miRNAs are simply due to reductions in OLs, as these miRNAs are highly expressed in mature OLs; single cell laser capture microdissection (LCM) may help to resolve this issue. Further studies comparing miRNA profiles of MS patient white matter to healthy control tissue has demonstrated an upregulation in miR-219 and -338 in MS patients (Noorbakhsh et al., 2011), suggesting an ongoing remyelination process in the MS brain. Demyelinated hippocampal sections of MS patients display reductions miR-138 expression (Dutta et al., 2013). These initial studies offer a gross examination of miRNA dysregulation in MS lesions, however further identification of miRNAs that have differential expression in cells of OL lineage within pathological states is still needed.

Perinatal hypoxia-ischemia (HI) in pre-term babies often results in white matter injury due to OPC susceptibility. Perinatal HI results in increased OPCs accompanied by reductions in mature OLs, suggesting a block in the transition from OPC to OL. The role of miRNAs in HI was investigated utilizing NG2 specific *Dicer1* KO mice. This model demonstrated that a loss of *Dicer1* in OPCs increased numbers of mature OLs and MBP expression in a murine model of HI (Birch et al., 2014). This increase in myelination was coupled with a recovery in murine motor performance. Furthermore, miRNA profiling in this murine model at multiple time points demonstrated increases in miR-138, -338, and -21 following HI (Birch et al., 2014). These results are unexpected due to impaired remyelination seen in *Dicer1* KO mice, along with the increases of miR-138 and -338 in OPC differentiation, an impaired process in HI. Further studies are needed to reconcile the roles of miRNAs in this disorder.

### miR-23a

miR-23a was initially identified as a regulator of lamin B1, an inhibitor of myelin gene expression (Lin and Fu, 2009). miR-23a directly represses lamin B1 and is inversely correlated with lamin B1 expression in mouse brain tissue; increased miR-23a expression promotes CNP and MBP expression in mixed glial cultures (Lin and Fu, 2009). Further work by this group has utilized CNP specific overexpression of miR-23a to assess the role of miR-23a in myelination (Lin et al., 2013) and demonstrated that OL specific overexpression of miR-23a results in significant hind-limb paralysis, loss of extensor tone and kyphosis compared to wildtype controls. The motor deficits observed in the miR-23a conditional knockout mice were accompanied by hypermyelination (which can also result in severe motor impairment) and increases in Luxol fast blue staining, as well as increased CNP, MBP, and MAG protein expression. Axons within the transgenic mice also demonstrated increases in myelin diameter observed by electron microscopy compared to controls. The *in vivo* changes in myelination were further corroborated by increases in OL cell number and myelin gene expression *in vitro*. The mechanism of action that underlies the phenotypic changes associated with increased miR-23a expression were linked to repression of the *Pten* gene, a known regulator of myelination that acts to inhibit Akt-signaling and growth factor signaling such as IGF-1 (Harrington et al., 2010; De Paula et al., 2014). Additionally, a long non-coding RNA (2700046G09Rik) was also identified as a novel regulator of OL development and is a bona fide miR-23a regulated gene (Lin et al., 2013).

Pathological studies of miRNA expression within the MS brain have compared miRNAs in both acute and chronic lesions, and the normal appearing white matter (NAWM; Junker et al., 2009). miRNA analysis of MS patient brain tissue demonstrated increases in miR-23a within active MS lesions. Furthermore, expression levels of miR-23a were dramatically increased in chronic lesions. miR-23a levels are also elevated in the spinal cords of EAE mice and non-human primates (Lescher et al., 2012). An interesting genetic analysis has demonstrated increases in the frequency of the miR-23a allele 3745453 have been identified as a risk factor in MS (Ridolfi et al., 2013). Increased miR-23a, a myelin promoting miRNA, in MS brain tissue may be the result of the ongoing remyelination process in MS, however, MS patient peripheral immune cells also demonstrate increased miR-23a expression (Ridolfi et al., 2013). Thus, the increases in miR-23a within the MS brain may be a byproduct of increased immune cell infiltration.

Leukodystrophies are diseases that display prominent loss of myelin in the absence of axonal loss and inflammation. Autosomal dominant leukodystrophy (ADLD) has been attributed to mutations in the *Lmnb1* gene that result in increased *Lmnb1* expression and myelin gene repression. As previously mentioned, miR-23a has been identified as a bona fide regulator of *Lmnb1*; increases in miR-23a can rescue deficits generated by *Lmnb1* overexpression *in vitro* (Lin and Fu, 2009). Further reports utilizing *in vivo* ADLD mouse models and patient samples are needed to further assess the role of miR-23a in this disease.

### miR-17-92 cluster

Analysis of miRNA expression patterns in cells along the OL lineage has demonstrated that several members of the miR-17-92 cluster are preferentially upregulated in OLs. Conditional deletion of the miR-17-92 cluster in CNP-expressing OLs diminished numbers of Olig2^+^ OLs. Furthermore, the addition of miR-17-92 cluster pre-miRNA leads to increased numbers of OLs *in vitro*; knockdown of pre-miR-17-92 decreased OL numbers (Budde et al., 2010). These changes were associated with regulation of *Pten* (Harrington et al., 2010), a regulator of OPC differentiation mentioned previously. The miR-17-92 cluster is also enriched in human white matter, suggesting that this cluster performs similar functions in the development of human oligodendrocytes. Despite this enrichment, the miR-17-92 cluster has reduced expression during the differentiation of rodent OPCs, a finding not replicated in human progenitor cells (de Faria et al., 2012).

### Sfmbt2 cluster

The *Sfmbt2* cluster is a cluster of miRNAs named after their distribution along the *Sfmbt2* gene, is expressed within embryonic stem cells, and impacts embryonic development (Zheng et al., 2011). This cluster of miRNAs has been recently identified as a regulator of oligodendrocyte development and remyelination during cuprizone-induced demyelination (Kuypers et al., 2016). Using the cuprizone model of demyelination (and subsequent remyelination), the authors used CNPase-eGFP reporter mice to identify cells of oligodendrocyte lineage. eGFP expressing cells were sorted from the corpus callosum of mice at multiple time points throughout the experimental timeline, and 38 miRNAs, including members of the *Sfmbt2* family, were downregulated during demyelination and subsequently increased during remyelination. Further analysis identified one miRNA, miR-297c-5p, as highly upregulated during oligodendrocyte differentiation. The functional significance of miR-297c-5p upregulation was attributed to the gene target Cyclin T2 (*Ccnt2*), a regulator of cell cycle progression and a negative regulator of OPC differentiation (Kim et al., 2012; Kuypers et al., 2016).

### Other miRNAs

miR-9 plays a critical role in the maturation of oligodendrocytes and Schwann cells by repressing PMP-22, a peripheral myelin protein (Adlkofer et al., 1995). miR-9 is enriched within OPCs of the CNS, but absent within the PNS, an expression pattern reciprocal with PMP-22 expression in these two compartments (Lau et al., 2008). Interestingly, PMP-22 mRNA is present within the CNS, however, its protein expression is absent (Dugas et al., 2006). These observations suggest that miR-9 may have additional roles in myelination of the CNS through the silencing of PNS myelin genes that would otherwise be inappropriately expressed within the CNS (Lau et al., 2008; Dugas and Notterpek, 2011); in the PNS, low levels of mir-9 may help to promote myelination by Schwann cells. When comparing miRNA levels in the MS brain, miR-9 is highly upregulated in chronic lesions vs. NAWM (Junker et al., 2009), however miR-9 levels are decreased compared to white matter from MS samples and healthy controls (Noorbakhsh et al., 2011).

miR-124 is highly expressed in mature neurons and influences the differentiation of progenitor cells to neurons. Despite its restricted expression in neurons, and not oligodendrocytes, miR-124 is increased during hippocampal demyelination in both human MS samples and mice undergoing cuprizone-induced demyelination (Dutta et al., 2013). miR-124 was also demonstrated to target AMPA receptor mRNA and potentially modulate learning and memory through this mechanism. This finding adds further complexity to the roles of miRNAs in myelin biology, as demyelination results in altered neural miRNA expression and subsequent function as a result of miRNA dysregulation. Furthermore, loss of miR-124 in zebrafish development resulted in reduced MBP^+^ cell numbers, likely due to the indirect influence of miR-124 on neurons and disruption of axonal-glial signals and cross-talk that are required for proper OL maturation and myelination (Morris et al., 2015).

Investigating the expression of miRNAs involved in OPC differentiation *in vitro* utilizing human embryonic stem cells has uncovered a handful of miRNAs that may be important in OL development. Validated miRNAs from this study include miR-199a-5p, -145, -214-, -184, and -1183 (Letzen et al., 2010). Predicted targets of these miRNAs include a variety of myelin-associated proteins, and specifically miR-214 is a likely regulator of myelin-associated oligodendrocyte basic protein that correlates with decreased expression of miR-214 during OPC development in primary human OPCs and embryonic stem cell derived progenitors (Letzen et al., 2010; Leong et al., 2014). miR-214 is also elevated in active and chronic MS lesions, potentially as a result of the ongoing remyelination process (Junker et al., 2009).

mir-146a is a well-characterized mediator of the innate immune response by targeting the *TRAF6* and *IRAK1* genes (Taganov et al., 2006). Using *in vitro* cultures of OPCs, the transfection of mature miR-146a promotes the differentiation of MBP expressing OLs from OPCs (Santra et al., 2014; Liu et al., 2016). *In situ* analysis using a rat model of stroke also identified increased miR-146a in the post-stroke brain. Increased expression of miR-146a was sufficient to increase the number of MBP+ OLs in an *in vitro* OPC culture system (Liu et al., 2016).

miRNAs within the miR-181 family have been identified as miRNAs with possible roles in OL biology. Specifically, miR-181a-1 is upregulated in the brains of mice lacking functional Dicer within PLP expressing OLs (Shin et al., 2009) while miR-181a, -181a*, -181c, and -181d were increased in OLs compared to OPCs (Dugas et al., 2010). miR-181a is additionally upregulated in isolated human fetal OPCs and adult OLs, however, expression is reduced in adult human OPCs and neural progenitors (Leong et al., 2014). The miR-181 family has previously been implicated in CNS ischemia and inflammation (Hutchison et al., 2013; Moon et al., 2013), although a functional role for this miRNA in OL differentiation has not yet been established. Interestingly, miR-181c has been implicated as a potential MS biomarker and is downregulated in the cerebrospinal fluid (CSF) of MS patients (Haghikia et al., 2012).

## miRNAs as potential biomarkers and therapeutic targets

miRNA expression patterns have been extensively studied as potential biomarkers in demyelinating diseases such as MS. With respect to inflammation and immune cell activity, potential miRNA biomarkers have been assessed in the peripheral compartment and include the miR-145 and -155 (Keller et al., 2009; Moore et al., 2013). Of interest to myelin biology, miR-92a-1, a member of the miR-17-92 cluster, has been identified as a potential biomarker in serum that is not only associated with MS patient disease severity and duration, but is also elevated in secondary progressive vs. relapsing MS (Gandhi et al., 2013). Serum levels of miR-23a are also downregulated in MS patients with primary progressive MS, a result that correlates with disability score (Fenoglio et al., 2013). The ability to identify decreases in myelin promoting miRNAs in the serum of patients undergoing progressive demyelination (a finding that correlated with disease score) may point to deficits in remyelination and the miRNAs underlying this process.

Beyond biomarkers, there has been an increasing interest in utilizing miRNAs as therapeutic targets in many disease states, including demyelinating diseases. The production of novel miRNA therapeutics typically seeks to either antagonize specific miRNAs that contribute to pathology, an approach taken with miR-122 in the course of Hepatitis C (Lanford et al., 2010), or administer exogenous miRNAs that promote resolution and repair. In the context of myelination and myelin repair, miR-219 has emerged as a potential target to enhance myelination. It has been demonstrated that exposure of aging animals to a youthful milieu is beneficial in promoting remyelination (Ruckh et al., 2012; Miron et al., 2013). Work by the Kraig laboratory has demonstrated that youthful rats and rats exposed to environmental enrichment generate serum exosomes that promote remyelination both *in vitro* utilizing lysolecithin treated slice cultures and *in vivo* following nasal administration of exosomes to aged rats (Pusic and Kraig, 2014). This effect was attributed to the presence of functional miR-219 within the exosomes, which was able to repress its target genes *NeuroD1, Pdgfr-*α, and *Elovl7*. Furthermore, IFNγ-stimulated dendritic cells were also shown to generate exosomes containing miR-219, which subsequently improved remyelination (Pusic et al., 2014).

## Discussion and perspectives

miRNAs have emerged as indispensable regulators of myelination of the CNS. Recent studies have characterized the roles of many key miRNA molecules that are central in this process and the mechanisms though which they exude their effect. However, several major areas of research still need to be addressed. First, although many of these molecules have been assessed in the context of development, research into the roles miRNAs perform in the remyelination process is needed. Secondly, the expression and possible dysregulation of miRNAs within the OL compartment must be assessed in the context of the pathological brain. Can we identify altered miRNA expression in OLs of individuals suffering from demyelinating disorder? Are there perhaps novel miRNA regulators that govern remyelination in the adult CNS? The use of *in vitro* and *in vivo* models of remyelination will be key in answering these questions. As miRNAs continue to move from the bench and into clinical trials, the possibility for miRNAs to prevent demyelination and promote remyelination represents a promising area in research and medicine.

## Author contributions

DG performed the literature search and drafted the manuscript. CM contributed by preparing the manuscript, editing, and submitting.

## Funding

CM laboratory is supported by grants from the Canada Research Chair program, Memorial University Faculty of Medicine, Research and Development Corporation of Newfoundland and Labrador, and the MS Society of Canada.

### Conflict of interest statement

The authors declare that the research was conducted in the absence of any commercial or financial relationships that could be construed as a potential conflict of interest.
